# Elacestrant in ER^+^, HER2^−^ Metastatic Breast Cancer with *ESR1*-Mutated Tumors: Subgroup Analyses from the Phase III EMERALD Trial by Prior Duration of Endocrine Therapy plus CDK4/6 Inhibitor and in Clinical Subgroups

**DOI:** 10.1158/1078-0432.CCR-24-1073

**Published:** 2024-08-01

**Authors:** Aditya Bardia, Javier Cortés, François-Clément Bidard, Patrick Neven, José Garcia-Sáenz, Phillipe Aftimos, Joyce O’Shaughnessy, Janice Lu, Giulia Tonini, Simona Scartoni, Alessandro Paoli, Monica Binaschi, Tomer Wasserman, Virginia Kaklamani

**Affiliations:** 1 University of California Los Angeles (UCLA) Health Jonsson Comprehensive Cancer Center, UCLA, Los Angeles, California.; 2 International Breast Cancer Center (IBCC), Pangaea Oncology, Quironsalud Group, Barcelona, Spain; and IOB Madrid, Hospital Beata Maria Ana, and Faculty of Biomedical and Health Sciences, Department of Medicine, Universidad Europea de Madrid, Madrid, Spain.; 3 Institut Curie, Paris and Saint Cloud, France.; 4 Universitaire Ziekenhuizen (UZ)—Leuven Cancer Institute, Leuven, Belgium.; 5 Instituto de Investigación Sanitaria Hospital Clinico San Carlos (IdISSC), Madrid, Spain.; 6 Institut Jules Bordet—Université Libre de Bruxelles, Brussels, Belgium.; 7 Baylor University Medical Center, Texas Oncology, US Oncology, Dallas, Texas.; 8 Northwestern University Robert H. Lurie Comprehensive Cancer Center, Chicago, Illinois.; 9 Menarini Group, Florence, Italy.; 10 University of Texas Health Sciences Center, San Antonio, Texas.

## Abstract

**Purpose:**

Elacestrant significantly prolonged progression-free survival (PFS) with manageable safety versus standard-of-care (SOC) endocrine therapy (ET) in patients with estrogen receptor–positive (ER^+^), HER2^−^ metastatic breast cancer and tumors harboring estrogen receptor 1 (*ESR1*) mutation following ET plus a cyclin-dependent kinase 4/6 inhibitor (ET+CDK4/6i). In patients with *ESR1*-mutated tumors, we evaluated the efficacy and safety of elacestrant versus SOC based on prior ET+CDK4/6i duration and in clinical subgroups with prior ET+CDK4/6i ≥12 months.

**Patients and Methods:**

EMERALD, an open-label phase III trial, randomly assigned patients with ER^+^, HER2^−^ metastatic breast cancer who had received 1–2 prior lines of ET, mandatory CDK4/6i, and ≤1 chemotherapy to elacestrant (345 mg daily) or SOC (aromatase inhibitor or fulvestrant). PFS was assessed across subgroups in *post hoc* exploratory analyses without adjustment for multiple testing.

**Results:**

In patients with *ESR1*-mutated tumors and prior ET+CDK4/6i ≥12 months, the median PFS for elacestrant versus SOC was 8.6 versus 1.9 months (HR, 0.41; 95% confidence interval, 0.26–0.63). In this population, the median PFS (in months) for elacestrant versus SOC was 9.1 versus 1.9 (bone metastases), 7.3 versus 1.9 (liver and/or lung metastases), 9.0 versus 1.9 (<3 metastatic sites), 10.8 versus 1.8 (≥3 metastatic sites), 5.5 versus 1.9 (*PIK3* catalytic subunit α mutation), 8.6 versus 1.9 (tumor protein p53 gene mutation), 9.0 versus 1.9 (HER2-low), 9.0 versus 1.9 (*ESR1*^D538G^-mutated tumors), and 9.0 versus 1.9 (*ESR1*^Y537S/N^-mutated tumors). Subgroup safety was consistent with the overall population.

**Conclusions:**

The duration of prior ET+CDK4/6i ≥12 months in metastatic breast cancer was associated with a clinically meaningful improvement in PFS for elacestrant compared with SOC and was consistent across all subgroups evaluated in patients with ER^+^, HER2^−^, *ESR1*-mutated tumors.

**Translational Relevance**

The phase III EMERALD trial demonstrated that single-agent elacestrant significantly prolonged progression-free survival (PFS) versus standard-of-care (SOC) endocrine monotherapy in patients with estrogen receptor–positive, HER2^−^ metastatic breast cancer who have been previously treated with endocrine therapy plus a CDK4/6 inhibitor (ET+CDK4/6i) and had estrogen receptor 1 (*ESR1*)–mutated tumors. *Post hoc*, exploratory subgroup analyses of EMERALD suggest that prior ET+CDK4/6i ≥12 months in metastatic breast cancer was associated with a clinically meaningful improvement in PFS for elacestrant versus SOC. Among patients with prior ET+CDK4/6i ≥12 months and *ESR1*-mutated tumors, elacestrant was associated with prolonged PFS versus SOC across relevant subgroups, regardless of metastatic site location or number, coexisting *PIK3* catalytic subunit α or tumor protein p53 gene mutations, HER2-low expression, or *ESR1* mutation variant. Prior ET+CDK4/6i ≥12 months may help identify patients with *ESR1*-mutated tumors that remain endocrine-sensitive to elacestrant, enabling ET sequencing in the second line before other targeted therapies and drug combinations, and may delay chemotherapy-based regimens, including antibody–drug conjugates.

## Introduction

The management of estrogen receptor–positive (ER^+^), HER2^−^ metastatic breast cancer involves endocrine therapy plus a cyclin-dependent kinase 4/6 inhibitor (ET+CDK4/6i) as the first-line standard-of-care (SOC) regimen (1–4).

The challenge of treating ER^+^, HER2^−^ metastatic breast cancer after first-line ET+CDK4/6i is to overcome endocrine resistance (5, 6). Molecular resistance patterns include intrinsic alterations of the PI3K/AKT/mTOR pathways, among others, and acquired resistance mechanisms (7–9). A common type of acquired resistance mechanism consists of alterations in the estrogen receptor 1 (*ESR1*) gene (7, 8, 10, 11). *ESR1* mutations occur in up to 50% of patients and predominantly emerge in the metastatic setting during first-line ET, particularly with aromatase inhibitors (AI; refs. 12–14).

Elacestrant is the first oral selective estrogen receptor degrader (SERD) to demonstrate increased efficacy compared with SOC endocrine monotherapy in the randomized phase III EMERALD trial, particularly in tumors harboring *ESR1* mutations, leading to regulatory approvals in the United States and Europe for the treatment of postmenopausal women or adult men with ER^+^, HER2^−^, *ESR1*-mutated advanced or metastatic breast cancer with disease progression following at least one line of ET (15–17). In EMERALD, single-agent elacestrant significantly prolonged the median PFS (mPFS; 3.8 vs. 1.9 months with SOC) and reduced the risk of progression or death by 45% versus SOC in patients with ER^+^, HER2^−^ metastatic breast cancer previously treated with ET+CDK4/6i and who had *ESR1*-mutated tumors [HR, 0.55; 95% confidence interval (CI), 0.39–0.77; *P* = 0.0005; ref. 15]. The PFS Kaplan–Meier curves revealed an initial drop in both arms, highlighting possible endocrine resistance for some patients in the second- or third-line setting, but then clear separation of the curves in the endocrine-sensitive setting, suggesting a treatment benefit for elacestrant in patients who have ER-driven disease.

The effects of tumor metastasis sites and the coexistence of common genomic alterations or other molecular expressions on the efficacy of elacestrant are of continued interest to better define treatment selection. *ESR1* mutations are associated with visceral metastases and endocrine resistance (18–23). Intrinsic alterations like *PIK3* catalytic subunit α mutations (*PIK3CA*-mut) and tumor protein p53 gene mutations (*TP53*-mut) occur in approximately 30% to 40% of ER^+^ breast cancers and confer poor prognosis and treatment resistance (24–36). The coexistence of *PIK3CA* and *ESR1* mutations can be found in approximately 15% to 30% of patients with ER^+^, HER2^−^ metastatic breast cancer (18, 37). The coexistence of *TP53* and *ESR1* mutations has been reported in 8% to 15% of tumors in patients with hormone receptor–positive, HER2^−^ metastatic breast cancer previously treated with ET (38, 39). HER2-low expression (HER2 IHC score of 1+ or 2+ without amplification by *ISH*; ref. 40) is prevalent in up to 65% of hormone receptor-positive breast cancers (41–43). The difference in prognostic value between HER2-low versus HER2-zero expression in metastatic breast cancer is limited, and evidence indicates that HER2-low disease biology is primarily driven by hormone receptor expression (44).

Evaluating subgroups of patients according to prior ET+CDK4/6i duration, metastatic site, and the presence of common coexisting mutations or molecular expressions with *ESR1* may help identify tumors that remain endocrine-sensitive despite acquired resistance to previous ET and thus help support clinical treatment decisions. To accomplish these goals, as these analyses were not prespecified in the EMERALD protocol, we conducted two *post hoc* exploratory subgroup analyses.

## Patients and Methods

EMERALD was an international, multicenter, randomized, open-label phase III clinical trial comparing single-agent elacestrant with SOC. The methodology of this trial has been previously described (15, 45). Eligible patients were postmenopausal women or men ages 18 years or older with ER^+^, HER2^−^ advanced or metastatic breast cancer who had received one or two prior lines of ET for advanced disease and mandatory prior treatment with a CDK4/6i in combination with fulvestrant or an AI. Patients were permitted to have received one prior line of chemotherapy in the advanced or metastatic setting.

Patients were randomized 1:1 to receive elacestrant 345 mg (equivalent to 400 mg elacestrant hydrochloride; ref. 16) once daily or investigator’s choice of SOC endocrine monotherapy (fulvestrant, letrozole, anastrozole, or exemestane). Investigators were advised to select fulvestrant for patients who had not previously received fulvestrant and select an AI for patients who had progressed on fulvestrant. Stratification factors were (i) the presence of *ESR1* mutation detected in ctDNA (*ESR1* mutation detected vs. *ESR1* mutation not detected), (ii) prior treatment with fulvestrant (yes vs. no), and (iii) the presence of visceral metastases (yes vs. no).

Tumor assessments were performed every 8 weeks using CT or MRI. Adverse events (AE) were collected until 30 days after the last dose of the study drug. Treatment was continued until objective disease progression based on standard RECIST 1.1 (46). The primary study endpoints for EMERALD were blinded independent review committee–assessed PFS in patients with tumors harboring detectable *ESR1* mutations and blinded independent review committee–assessed PFS in all patients, regardless of tumor *ESR1* mutation status.

The EMERALD trial was conducted in accordance with ethical principles consistent with the Declaration of Helsinki and International Council of Harmonisation/Good Clinical Practice. The study protocol and relevant supporting information were approved by the institutional review board at each participating site, and each participant provided written informed consent.

### Study outcome measures

In the first subgroup analysis, PFS was assessed according to the prior duration of ET+CDK4/6i in the advanced or metastatic setting in patients receiving elacestrant versus SOC among those with tumors harboring detectable *ESR1* mutations. Considering that longer exposure to ET during the treatment of the metastatic disease is related to an increased risk of developing an *ESR1* mutation (7–9), patient subgroups were defined according to the duration of prior ET+CDK4/6i (≥6, ≥12, and ≥18 months).

In the second subgroup analysis, PFS was assessed in patients receiving elacestrant versus SOC with tumors harboring detectable *ESR1* mutations who had received prior ET+CDK4/6i ≥12 months in the advanced or metastatic setting and had at least one of the following covariates at screening: (i) the presence of bone metastasis; (ii) the presence of liver and/or lung metastasis; (iii) <3 or ≥3 metastatic sites; (iv) *PIK3CA*-mut as detected by ctDNA; (v) *TP53*-mut as detected by ctDNA; (vi) HER2-low tumor expression detected by IHC; or (vii) *ESR1* mutation variants D538G and Y537S/N.

Safety was assessed in the overall population (patients with and without *ESR1*-mutated tumors) according to the treatment arm and in patients with *ESR1*-mutated tumors by the treatment arm according to ET+CDK4/6i duration and clinically relevant subgroups among patients who had received prior ET+CDK4/6i ≥12 months.

### Statistical analysis

*Post hoc*, exploratory analyses were performed using Kaplan–Meier methods to estimate the survival distribution function of PFS, without adjustment for multiple testing. Analyses were based on the intention-to-treat population for patients with *ESR1*-mutated tumors. HRs and 95% CIs for elacestrant versus SOC were calculated using the Cox regression model stratified by randomization stratification factors, including treatment as a variable and the subgroups listed above as covariates. A landmark analysis was also performed, estimating PFS rates at 6, 12, and 18 months. All analyses were performed using SAS (SAS Institute).

### Data availability

Data that underlie the results reported in a published article may be requested for products and the relevant indications that have been authorized by the regulatory authorities in Europe/the United States (or, if not, data can be requested for up to 6 years after publication).

The Menarini Group will review requests individually to determine whether (1) the requests are legitimate and relevant and meet sound scientific research principles, (2) are within the scope of the participants’ informed consent, and (3) are compliant with any applicable law and regulation and with any contractual relationship that the Menarini Group, its affiliates, and partners have in place with respect to the study and/or the relevant product. Before making data available, requestors will be required to agree in writing to certain obligations, including without limitation, compliance with applicable privacy, and other laws and regulations. Proposals should be directed to medicalinformation@menarinistemline.com.

## Results

A total of 478 patients were randomized in EMERALD (elacestrant, *n* = 239; SOC, *n* = 239; Supplementary Fig. S1). Of these, 222 patients (elacestrant, *n* = 112; SOC, *n* = 110) had *ESR1-*mutated tumors, had received their ET+CDK4/6i treatment in the advanced or metastatic setting, and were analyzed for PFS according to ET+CDK4/6i duration. Among this population, 159 patients (71.6%) received prior ET+CDK4/6i ≥12 months; these patients form the overall population of the clinical subgroup analysis. The baseline characteristics of this population (*n* = 159) are shown in Table 1. The representativeness of study participants is shown in Supplementary Table S1. Patient characteristics based on the duration of exposure to ET+CDK4/6i were generally well balanced between treatment arms within each subgroup (Supplementary Table S2).

**Table 1.** Baseline characteristics in patients with *ESR1*-mutated tumors and prior ET+CDK4/6i ≥12 months.

Parameter	Elacestrant (*N* = 78)	SOC (*N* = 81)
Median age, years (range)	65.5 (40–89)	63 (32–82)
Female, *n* (%)	78 (100)	81 (100)
Race or ethnicity, *n* (%)		
Asian	3 (3.9)	3 (3.7)
Black or African American	3 (3.9)	4 (4.9)
Other	1 (1.3)	0
White	59 (75.6)	59 (72.8)
Hispanic or Latino	6 (7.7)	7 (8.6)
ECOG PS 0, *n* (%)	42 (53.9)	49 (60.5)
Metastatic site, *n* (%)		
Bone^a^	67 (85.9)	69 (85.2)
Visceral	58 (74.4)	57 (70.4)
Liver and/or lung^b^	56 (71.8)	57 (70.3)
Number of metastatic sites, *n* (%)^c^		
<3	42 (53.8)	40 (49.4)
≥3	28 (35.9)	25 (30.9)
Mutations, *n* (%)		
*ESR1*^d^	78 (100)	81 (100)
D538G	48 (61.5)	49 (60.5)
Y537S/N	49 (62.8)	43 (53.1)
*PIK3CA*^e^	27 (34.6)	35 (43.2)
H1047X	10 (12.8)	16 (19.8)
E542X and E545X	12 (15.4)	15 (18.5)
*TP53*	32 (41.0)	29 (35.8)
*BRCA1/2*	16 (20.5)	16 (19.8)
HER2-low expression^f^	37 (47.4)	40 (49.4)
Prior adjuvant therapy, *n* (%)	44 (56.4)	47 (58.0)
No. of prior lines of ET in the advanced or metastatic setting, *n* (%)		
1	49 (62.8)	55 (67.9)
2	29 (37.2)	26 (32.1)
No. of prior lines of chemotherapy in the advanced or metastatic setting, *n* (%)		
0	62 (79.5)	63 (77.8)
1	16 (20.5)	18 (22.2)
Prior CDK4/6i, *n* (%)		
Abemaciclib	3 (3.8)	3 (3.7)
Palbociclib	70 (89.7)	77 (95.1)
Ribociclib	14 (17.9)	11 (13.6)
Any prior ET, *n* (%)	78 (100)	80 (98.8)
Fulvestrant, *n* (%)	13 (16.7)	22 (27.2)
AI, *n* (%)	72 (92.3)	71 (87.7)
Tamoxifen, *n* (%)	7 (9.0)	7 (8.6)
PI3K inhibitor, *n* (%)	0	0
mTOR inhibitor, *n* (%)	5 (6.4)	1 (1.2)

Abbreviations: *BRCA1/2*, breast cancer gene 1 and/or 2; ECOG PS, Eastern Cooperative Oncology Group performance status.

a Eighty-five percent of patients had bone and other sites of metastases (30% of these patients had no liver or lung involvement).

b Fifty-five percent of patients had liver and other sites of metastases (10% of these patients had no lung or bone involvement); 25% of patients had lung and other sites of metastases (2% of these patients had no liver or bone involvement).

c The number of metastatic sites was available for 135 of 159 patients with *ESR1*-mutated tumors and prior ET+CDK4/6i ≥12 months.

d Ninety percent of patients had one or more *ESR1* mutations detected in the three hot spots presented (D538G, Y537S, and/or Y537N).

e Includes E545K, H1047R, E542K, and others.

f Locally assessed HER2 IHC score of 1+ and 2+ with no ISH amplification. Data not available for all patients.

### PFS by ET+CDK4/6i duration

**Figure 1.**
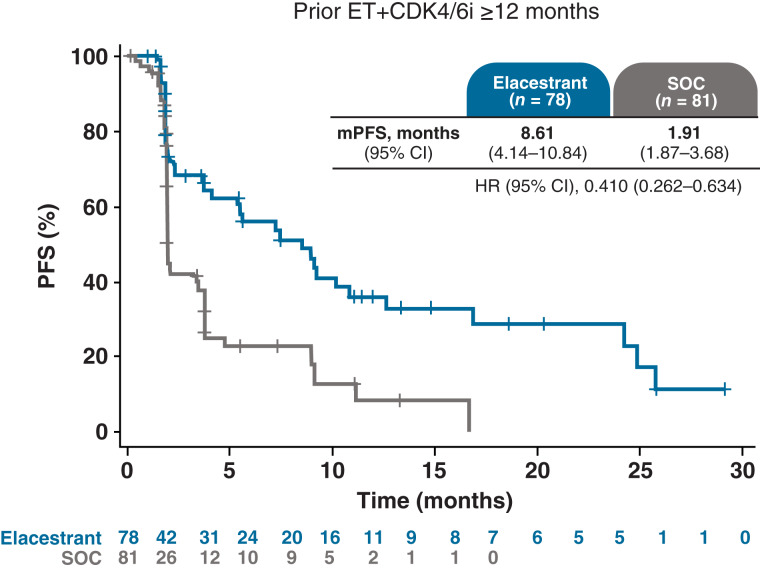
PFS in patients who received prior ET+CDK4/6i ≥12 months in the metastatic setting. Kaplan–Meier estimates of PFS in patients with *ESR1*-mutated tumors and prior ET+CDK4/6i ≥12 months in the metastatic setting (*n* = 159).

A longer duration of prior ET+CDK4/6i therapy was associated with a clinically meaningful improvement in PFS for elacestrant compared with SOC in patients with *ESR1*-mutated tumors. In patients with prior ET+CDK4/6i ≥12 months, the mPFS with elacestrant was 8.6 versus 1.9 months with SOC (HR, 0.41; 95% CI, 0.26–0.63; Fig. 1). The *P* value for interaction between elacestrant treatment and prior ET+CDK4/6i duration (<12 vs. ≥12 months) was statistically significant (*P* = 0.014). An improvement in PFS was also associated with elacestrant compared with SOC in patients with prior ET+CDK4/6i ≥6 and ≥18 months (Supplementary Fig. S2). Elacestrant was associated with a clinical benefit in all subgroups, with the magnitude of PFS improvement greater in patients who received prior ET+CDK4/6i ≥12 months. In those patients who received fulvestrant, the mPFS ranged from 1.9 to 2.1 months across the subgroups evaluated based on prior ET+CDK4/6i duration (Supplementary Fig. S3).

### PFS in clinically relevant subgroups

**Figure 2.**
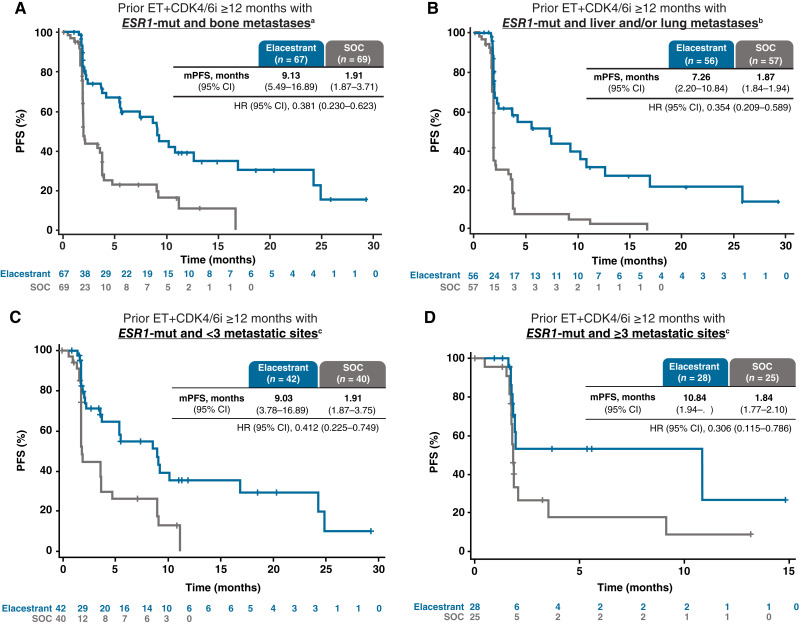
PFS according to clinical subgroups. Kaplan–Meier estimates of PFS in patients with *ESR1*-mutated tumors and prior ET+CDK4/6i ≥12 months who, at screening, had the presence of (**A**) bone metastases (*n* = 136; 86%); (**B**) liver and/or lung metastases (*n* = 113; 71%); (**C**) <3 metastatic sites (*n* = 82; 52%); or (**D**) ≥3 metastatic sites (*n* = 53; 33%). ^a^Eighty-five percent of patients had bone and other sites of metastases (30% of these patients had no liver or lung involvement). ^b^Fifty-five percent of patients had liver and other sites of metastases (10% of these patients had no lung or bone involvement); 25% of patients had lung and other sites of metastases (2% of these patients had no liver or bone involvement). ^c^The number of metastatic sites was available for 135 of 159 patients with *ESR1*-mutated tumors and prior ET+CDK4/6i ≥12 months.

**Figure 3.**
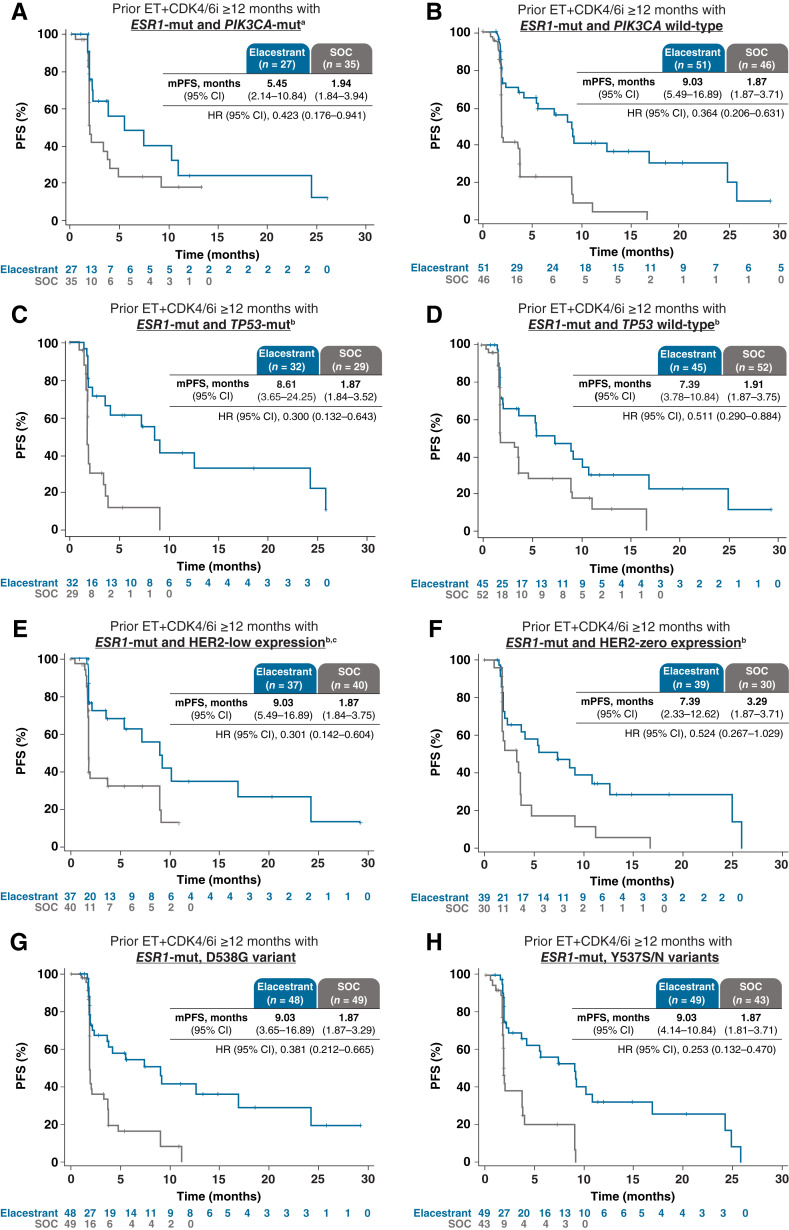
PFS according to mutation and molecular expression subgroups. Kaplan–Meier estimates of PFS in patients with *ESR1*-mutated tumors and prior ET+CDK4/6i ≥12 months who, at screening, had the presence of (**A**) *PIK3CA*-mut (*n* = 62; 39%); (**B**) *PIK3CA* wild-type (*n* = 97; 61%); (**C**) *TP53*-mut (*n* = 61; 38%); (**D**) *TP53* wild-type (*n* = 97; 61%); (**E**) HER2-low expression (*n* = 77; 48%); (**F**) HER2-zero expression (*n* = 69; 43%); (**G**) *ESR1*^D538G^-mutated tumors (*n* = 97; 61%); or (**H**) *ESR1*^Y537S/N^-mutated tumors (*n* = 92; 58%). ^a^Includes the following *PIK3CA*-mut: E545K, H1047R, and E542K among others. ^b^Data not available for all patients. ^c^HER2-low expression defined as IHC score of 1+ or 2+ with no amplification by ISH.

Across all subgroups evaluated, a clinically meaningful improvement in PFS was associated with elacestrant compared with SOC in those patients with *ESR1*-mutated tumors who received prior ET+CDK4/6i ≥12 months, regardless of the metastatic site location or number; coexistence of *PIK3CA*-mut, *TP53*-mut, or HER2-low expression; or *ESR1* mutation variant (Table 2; Figs. 2 and 3). Among patients with bone metastases and *ESR1*-mutated tumors, the mPFS with elacestrant was 9.1 versus 1.9 months with SOC (HR, 0.38; 95% CI, 0.23–0.62). Among patients with liver and/or lung metastases and *ESR1*-mutated tumors, the mPFS with elacestrant was 7.3 versus 1.9 months with SOC (HR, 0.35; 95% CI, 0.21–0.59). Among patients with <3 metastatic sites and *ESR1*-mutated tumors, the mPFS with elacestrant was 9.0 versus 1.9 months with SOC (HR, 0.41; 95% CI, 0.23–0.75). Among patients with ≥3 metastatic sites and *ESR1*-mutated tumors, the mPFS with elacestrant was 10.8 versus 1.8 months with SOC (HR, 0.31; 95% CI, 0.12–0.79). Among patients with *PIK3CA*- and *ESR1*-mutated tumors, the mPFS with elacestrant was 5.5 versus 1.9 months with SOC (HR, 0.42; 95% CI, 0.18–0.94). Among patients with *TP53*-mutated and *ESR1*-mutated tumors, the mPFS with elacestrant was 8.6 versus 1.9 months with SOC (HR, 0.30; 95% CI, 0.13–0.64). Among patients with HER2-low tumor expression and *ESR1-*mutated tumors, the mPFS with elacestrant was 9.0 versus 1.9 months with SOC (HR, 0.30; 95% CI, 0.14–0.60). Among patients with *ESR1*^D538G^-mutated tumors, the mPFS with elacestrant was 9.0 versus 1.9 months with SOC (HR, 0.38; 95% CI, 0.21–0.67). Among patients with *ESR1*^Y537S/N^-mutated tumors, the mPFS with elacestrant was 9.0 versus 1.9 months with SOC (HR, 0.25; 95% CI, 0.13–0.47). *P* values for interaction between elacestrant treatment and the following variables suggest that the presence of these coexisting mutations or molecular expressions did not impact the benefit observed with elacestrant versus SOC: *PIK3CA*-mut (*P* = 0.13), *TP53*-mut (*P* = 0.47), and HER2-low expression (*P* = 0.32). A similar benefit was associated with elacestrant when analyzed by *PIK3CA*-mut locations and *BRCA1/2* mutation (Supplementary Table S3).

**Table 2.** PFS in subgroups of patients with *ESR1*-mutated tumors and prior ET+CDK4/6i ≥12 months.

Patient subgroup	*n* (%)	mPFS, months		HR (95% CI)
		Elacestrant	SOC	
All patients with *ESR1-*mutated tumors	159 (100)	8.6	1.9	0.41 (0.26–0.63)
*ESR1-*mutated tumors and bone metastases^a^	136 (86)	9.1	1.9	0.38 (0.23–0.62)
*ESR1*-mutated tumors and liver and/or lung^b^ metastases	113 (71)	7.3	1.9	0.35 (0.21–0.59)
*ESR1-*mutated tumors and <3 metastatic sites^c^	82 (52)	9.0	1.9	0.41 (0.23–0.75)
*ESR1-*mutated tumors and ≥3 metastatic sites^c^	53 (33)	10.8	1.8	0.31 (0.12–0.79)
*ESR1-* and *PIK3CA*-mutated tumors^d^	62 (39)	5.5	1.9	0.42 (0.18–0.94)
*ESR1-* and *TP53*-mutated tumors	61 (38)	8.6	1.9	0.30 (0.13–0.64)
*ESR1-*mutated tumors and HER2-low expression^e^	77 (48)	9.0	1.9	0.30 (0.14–0.60)
*ESR1* ^D538G^ *-*mutated tumors	97 (61)	9.0	1.9	0.38 (0.21–0.67)
*ESR1* ^Y537S/N^ *-*mutated tumors	92 (58)	9.0	1.9	0.25 (0.13–0.47)

a Eighty-five percent of patients had bone and other sites of metastases (30% of these patients had no liver or lung involvement).

b Fifty-five percent of patients had liver and other sites of metastases (10% of these patients had no lung or bone involvement); 25% of patients had lung and other sites of metastases (2% of these patients had no liver or bone involvement).

c The number of metastatic sites was available for 135 of 159 patients with *ESR1-*mutated tumors and prior ET+CDK4/6i ≥12 months.

d Includes E545K, H1047R, E542K, and others.

e Locally assessed HER2 IHC score of 1+ and 2+ with no ISH amplification. Data not available for all patients.

### Safety

In the overall population (patients with or without *ESR1*-mutated tumors), the majority of AEs that occurred were of grade 1 or 2 severity, including nausea (15). Treatment discontinuations due to any treatment-related AE occurred in eight patients (3.4%) receiving elacestrant and two patients (0.9%) receiving SOC. No deaths assessed as treatment-related were reported in either arm. No hematologic safety signal was observed, and sinus bradycardia was not reported in either treatment arm.

Updated safety analysis of the most common AEs and detailed information about nausea and antiemetic use are outlined in Table 3. The most common all-grade gastrointestinal AEs observed were nausea (35% with elacestrant vs. 19% with SOC) and vomiting (19% with elacestrant vs. 9% with SOC). No patient experienced grade 4 nausea or vomiting with elacestrant. Both elacestrant dose-reduction and discontinuation rates due to nausea were 1.3%. Antiemetics were required by 8% of patients treated with elacestrant, 10.3% of patients with AIs, and 3.7% with fulvestrant. Safety data for patients with *ESR1*-mutated tumors by prior ET +CDK4/6i duration or clinical and biomarker subgroups were consistent with the profile in the overall population (Supplementary Tables S4–S15).

**Table 3.** Most common AEs (≥10% in either arm) in the overall population (16).

Adverse reaction^a^	Elacestrant (*n* =237)		SOC (*n* = 230)	
	All grades (%)	Grade ≥3 (%)	All grades (%)	Grade ≥3 (%)
Musculoskeletal and connective tissue disorders				
Musculoskeletal pain^b^	41	7	39	1
Gastrointestinal disorders				
Nausea	35	2.5	19	0.9
Vomiting^b^	19	0.8	9	0
Diarrhea	13	0	10	1
Constipation	12	0	6	0
Abdominal pain^b^	11	1	10	0.9
Dyspepsia	10	0	2.6	0
General disorders and administration site conditions				
Fatigue^b^	26	2	27	1
Metabolism and nutritional disorders				
Decreased appetite	15	0.8	10	0.4
Nervous system disorders				
Headache	12	2	12	0
Vascular disorders				
Hot flush	11	0	8	0

Nausea-related AEs in the overall population, *n* (%)				
Dose-reduction rate due to nausea	3 (1.3)		Not applicable	
Discontinuation rate due to nausea	3 (1.3)		0	
Antiemetic use	19 (8.0)		AI: 7 (10.3)Fulvestrant: 6 (3.7)	

a Adverse reactions were graded using NCI Common Terminology Criteria for Adverse Events version 5.0.

b Includes other related terms.

## Discussion

These subgroup analyses of EMERALD suggest that a longer duration of prior ET+CDK4/6i was associated with clinically meaningful improvement in PFS for elacestrant compared with SOC endocrine monotherapy in patients with *ESR1*-mutated, ER^+^, HER2^–^ metastatic breast cancer. In patients who had received prior ET+CDK4/6i ≥12 months, elacestrant was associated with an mPFS of 8.6 versus 1.9 months with SOC. The statistically significant *P* value for interaction between elacestrant treatment and prior CDK4/6i duration of <12 versus ≥12 months suggests that longer exposure to CDK4/6i is associated with endocrine sensitivity to elacestrant in *ESR1*-mutated tumors. Additional subgroup analyses suggest that among patients with *ESR1*-mutated tumors who received prior ET+CDK4/6i ≥12 months, single-agent elacestrant was associated with a prolonged PFS versus SOC for patients in clinically relevant subgroups, including patients with bone metastases, liver and/or lung metastases, <3 or ≥3 metastatic sites, or tumors with *PIK3CA*-mut, *TP53*-mut, HER2-low tumor expression, or *ESR1* mutation variants D538G or Y537S/N. *P* values for interaction between elacestrant treatment and *PIK3CA*-mut, *TP53*-mut, or HER2-low expression suggested that the benefit observed with elacestrant versus SOC was not impacted by the presence of these common coexisting mutations or molecular expressions.

Mutations of *ESR1* occur during exposure to ET in the metastatic setting, increasing to up to 50% after first-line treatment (14, 47). Based on this high rate and availability of an effective *ESR1*-targeting therapeutic, testing for the emergence of *ESR1* mutations at each disease progression is recommended by the National Comprehensive Cancer Network, American Society of Clinical Oncology, and European Society of Medical Oncology guidelines (2, 4, 48).

For patients with ER^+^, HER2^−^, *ESR1*-mutated metastatic breast cancer who had disease progression on prior ET+CDK4/6i, subsequent ET-based treatment options include endocrine monotherapy, continuation of ET+CDK4/6i, or PI3K/AKT/mTOR pathway–ET combination regimens. Although endocrine monotherapy is a well-tolerated treatment option, continuing AI monotherapy is limited by potential resistance in a population with *ESR1*-mutated tumors (49–52), and fulvestrant has been associated with an mPFS of approximately 2 to 3 months in the post-CDK4/6i and *ESR1* mutation setting (15, 53). The presence of acquired resistance mechanisms to conventional ET requires treatment options that target *ESR1* mutations. Oral SERDs other than elacestrant are in development; however, none of the clinical trials in later-line settings required prior CDK4/6i therapy for all participants, limiting the available information in this patient subset (54–59).

Continuing ET+CDK4/6i therapy is an alternative option to endocrine monotherapy. However, current evidence does not support this practice in patients with *ESR1*-mutated tumors (53, 60–62). The MAINTAIN trial demonstrated a 3-month mPFS with fulvestrant with or without ribociclib in this subgroup, and no benefit was observed in patients who received prior ET+CDK4/6i >12 months [*n* = 80 (67.2%); HR, 0.76; 95% CI, 0.47–1.24; ref. 53]. In PACE (prior ET+CDK4/6i >12 months, 76%), among patients with *ESR1*-mutated tumors (*n* = 78), palbociclib plus fulvestrant was associated with an mPFS of 5.2 months versus 3.3 months with fulvestrant alone (HR, 0.68; 95% CI, 0.42–1.09; ref. 61). In PALMIRA, patients with prior ET+CDK4/6i ≥12 months (*n* = 170, 85.9%) had an mPFS of 4.2 months with palbociclib plus ET versus 3.6 months for endocrine monotherapy (HR, 0.83; 95% CI, 0.63–1.07; *P* = 0.154); data on *ESR1*-mutated tumors were not reported (60). In postMONARCH, an mPFS of 6.0 months was observed for abemaciclib plus fulvestrant versus 5.3 months with fulvestrant plus placebo (HR, 0.73; 95% CI, 0.57–0.95; *P* = 0.02); the mPFS for patients with *ESR1*-mutated tumors was not reported (HR, 0.79; 95% CI, 0.54–1.15; ref. 62).

Data on PI3K/AKT/mTOR pathway inhibitors in patients with *ESR1*-mutated tumors who have received prior ET+CDK4/6i ≥12 months are not available. TRINITI-1 demonstrated an mPFS of 3.5 months with post-CDK4/6i everolimus plus exemestane plus ribociclib in patients with *ESR1*-mutated tumors (63). In BYLieve, which evaluated alpelisib plus ET in patients who had tumors harboring coexisting *PIK3CA* and *ESR1* mutations and had received an AI plus CDK4/6i, the mPFS ranged from 4.6 to 5.6 months (28, 64–65). In CAPItello-291, among patients with AKT pathway alterations who received prior ET+CDK4/6i, capivasertib plus fulvestrant was associated with an mPFS of 5.5 months versus 2.0 months with placebo plus fulvestrant; no data on *ESR1*-mutated tumors were reported (66, 67). Our findings suggest a clinical benefit with elacestrant in patients with tumors harboring coexisting *ESR1* and *PIK3CA*-mut, indicating that disease progression after ET+CDK4/6i in this subgroup may remain ER-driven. These analyses, together with our additional subgroup analyses by metastatic site location or number and in patients with *TP53*-mutated tumors, HER2-low tumor expression, or different *ESR1* mutation variants, suggest that elacestrant can be an option for patients with endocrine-sensitive tumors.

Safety analyses demonstrated that elacestrant had a manageable safety profile similar to other ETs and without evidence of some of the toxicities associated with other drug classes, such as CDK4/6i and PI3K/AKT/mTOR inhibitors. CDK4/6i combinations are associated with neutropenia, leukopenia, anemia, and diarrhea, with discontinuations due to AEs in up to 19% of patients (53, 68). The use of PI3K/AKT/mTOR pathway inhibitors plus ET is associated with diarrhea, rash, and hyperglycemia, resulting in discontinuations due to AEs in up to 24% of patients (64, 66, 69).

The findings from our analyses are hypothesis-generating due to their *post hoc* exploratory nature and may be used to help identify signals in patients with tumors that remain endocrine-sensitive. Our analyses provide additional evidence in clinically important subgroups of patients, representative of the current clinical setting in which patients have received prior ET+CDK4/6i ≥12 months. These analyses also provide evidence that may help inform real-world clinical decision-making in the second-line, post-ET+CDK4/6i setting for patients with tumors harboring *ESR1* mutation.

### Conclusions

These *post hoc* exploratory subgroup analyses suggest that a duration of prior ET+CDK4/6i ≥12 months was associated with a clinically meaningful improvement in PFS for elacestrant compared with SOC endocrine monotherapy in patients with ER^+^, HER2^−^ metastatic breast cancer and *ESR1*-mutated tumors. The PFS benefit associated with elacestrant was consistent across clinically relevant subgroups evaluated, including patients with bone metastases, liver and/or lung metastases, <3 or ≥3 metastatic sites, *PIK3CA*-mutated tumors, *TP53*-mutated tumors, HER2-low tumor expression, or *ESR1* mutation variants D538G or Y537S/N. Subgroup safety analyses demonstrated that elacestrant has a manageable safety profile that is consistent with the profile in the overall population. These data support current guidelines that recommend routine testing for the emergence of *ESR1* mutations in ctDNA at each disease progression. Although future studies are warranted, these results suggest that elacestrant may enable ET sequencing in the second line before other targeted therapies and drug combinations and may delay chemotherapy-based regimens, including antibody–drug conjugates.

## Supplementary Material

Supplementary Data1 Supplemental figures 1-3; Supplemental tables 1-15
