# Supplementary material for: Elacestrant in ER+, HER2− Metastatic Breast Cancer with ESR1-Mutated Tumors: Subgroup Analyses from the Phase III EMERALD Trial by Prior Duration of Endocrine Therapy plus CDK4/6 Inhibitor and in Clinical Subgroups
Source: Clin Cancer Res. 2024 Aug 1;30(19):4299–309. doi: 10.1158/1078-0432.CCR-24-1073 (PMC11443208; doi:10.1158/1078-0432.CCR-24-1073)
Supplement: Supplementary Data1 — Supplemental figures 1-3; Supplemental tables 1-15 [file ccr-24-1073_supplementary_data1_suppds1.pdf]

SUPPLEMENT

Contents

Figures 2
Tables 5

Figures

Figure S1: Patient Disposition

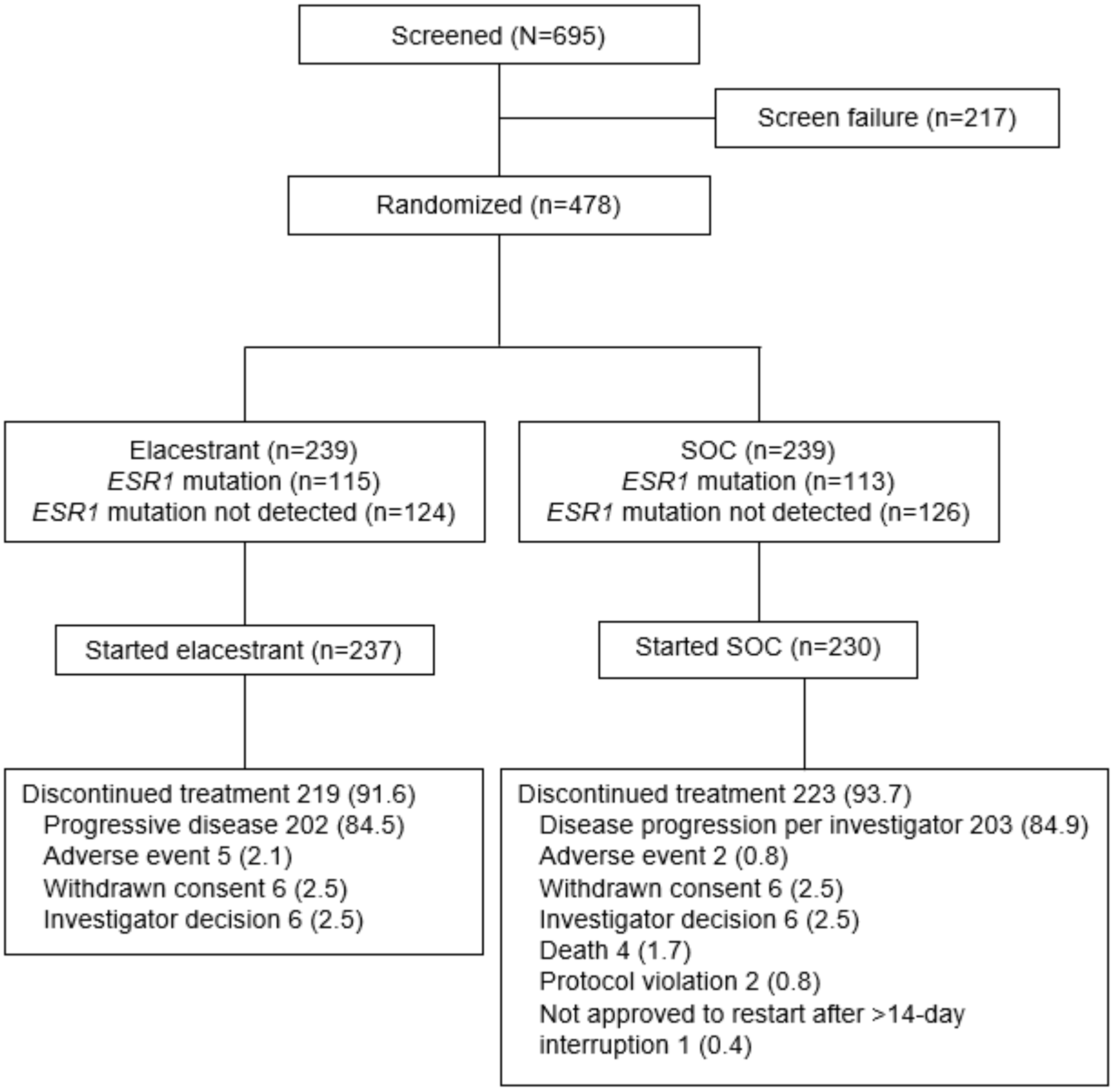

Of the 228 patients who had *ESR1*-mutated tumors (elacestrant, n=115; SOC, n=113), 222 patients had received their ET+CDK4/6i treatment in the advanced or metastatic setting (elacestrant, n=112; SOC, n=110) and were analyzed for PFS according to ET+CDK4/6i duration. ET+CDK4/6i, endocrine therapy plus cyclin-dependent kinase 4/6 inhibitor; *ESR1,* estrogen receptor 1; SOC, standard-of-care endocrine therapy (aromatase inhibitor or fulvestrant).

Figure S2. Kaplan-Meier Estimates of Progression-free Survival in Patients with *ESR1*-mutated Tumors Receiving Elacestrant or SOC, According to Prior ET+CDK4/6i ≥6, ≥12, or ≥18 Months in the Metastatic Setting

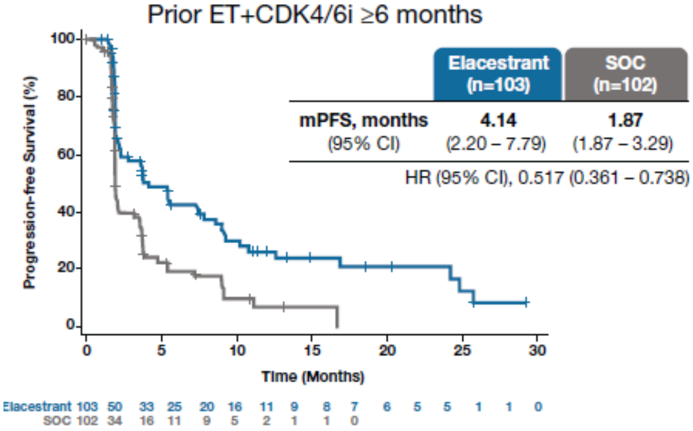

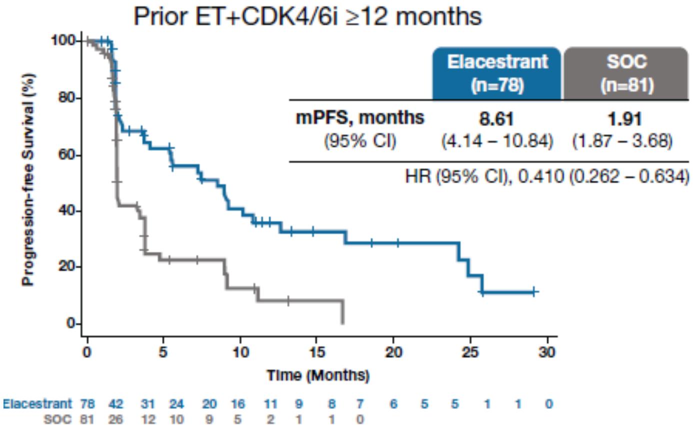

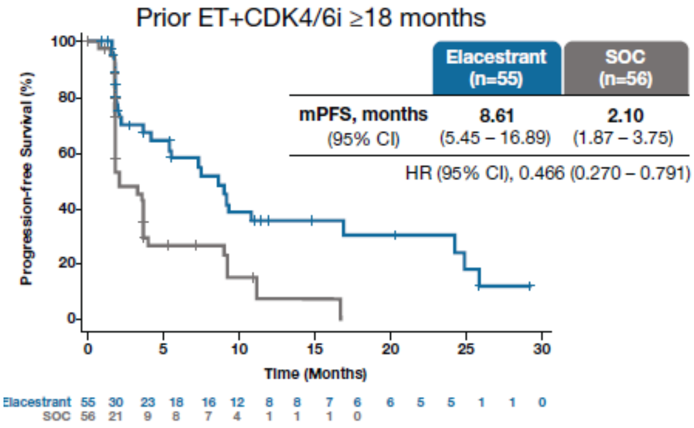

CI, confidence interval; *ESR1*, estrogen receptor 1; ET+CDK4/6i, endocrine therapy plus cyclin-dependent kinase 4/6 inhibitor; HR, hazard ratio; mPFS, median progression-free survival; SOC, standard-of-care endocrine therapy (aromatase inhibitor or fulvestrant).

Figure S3. Kaplan-Meier Estimates of Progression-free Survival in Patients with *ESR1*-mutated Tumors Receiving Elacestrant or Fulvestrant, According to Prior ET+CDK4/6i ≥6, ≥12, or ≥18 Months in the Metastatic Setting

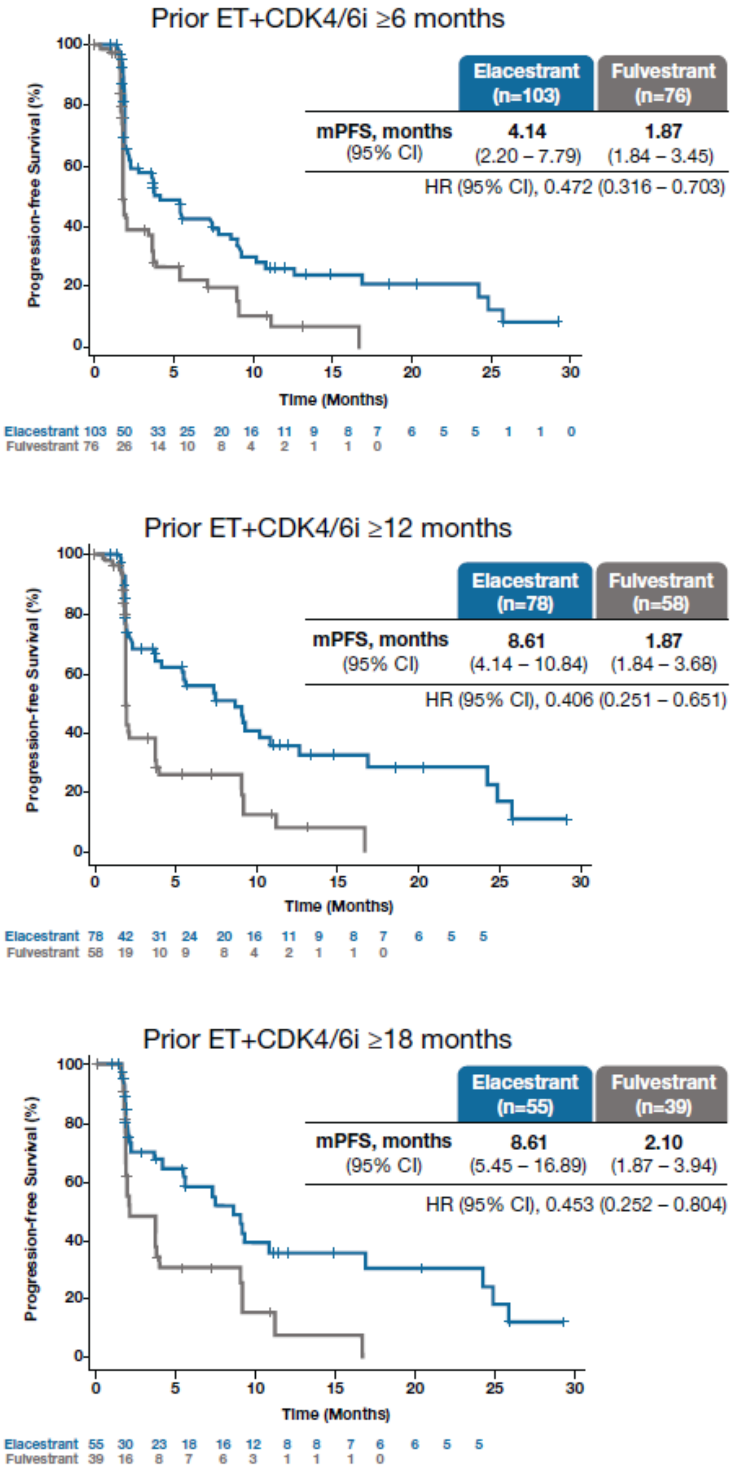

CI, confidence interval; *ESR1*, estrogen receptor 1; ET+CDK4/6i, endocrine therapy plus cyclin-dependent kinase 4/6 inhibitor; HR, hazard ratio; mPFS, median progression-free survival; SOC, standard-of-care endocrine therapy (aromatase inhibitor or fulvestrant).

Tables

Table S1. Representativeness of study participants

Cancer type(s)/subtype(s)/stage(s)/ condition	Hormone receptor-positive/human epidermal growth factor receptor 2-negative (HR+, HER2–) unresectable locally advanced not amenable to surgical resection or radiotherapy with curative intent or metastatic breast cancer.
Considerations related to:	
Sex	HR+, HER2– breast cancer, is a predominantly female disease (99%).<br>Men account for around 1% of all new diagnoses of this disease.[1]
Age	The median age at diagnosis for patients with metastatic breast cancer is 62-66 years.[1,2]
Race/ethnicity	White women are slightly more likely to develop breast cancer than Black, Hispanic, and Asian women.[3]
Geography	Breast cancer incidence:[4]<br>Asia = 1,026,171<br>Europe = 531,086<br>North America = 281,591<br>Latin America/Caribbean = 210,100<br>Africa = 186,598<br>Oceana = 25,873
Other Considerations	Breast cancer clinical trials historically underrepresent minorities, including Black patients relative to the overall US population
Overall representativeness of this study	Overall, the study representativeness aligns with the literature. The median age of patients in our study was 63-66 years. All patients were female, and the majority of patients were White followed by Hispanic, Black, and Asian. Patients were enrolled globally from Australia, Austria, Argentina, Belgium, Canada, Denmark, France, Greece, Hungary, Ireland, Israel, Italy, Korea, Portugal, Spain, UK, and USA

1. Meegdes M, Geurts SME, Erdkamp FLG, Dercksen MW, Vriens BEPJ, Aaldering KNA, et al. Real-world time trends in overall survival, treatments and patient characteristics in HR+/HER2− metastatic breast cancer: an observational study of the SONABRE Registry. The Lancet Regional Health – Europe 2023;26.
2. Chen MT, Sun HF, Zhao Y, Fu WY, Yang LP, Gao SP, et al. Comparison of patterns and prognosis among distant metastatic breast cancer patients by age groups: a SEER population-based analysis. Sci Rep 2017;7:9254.
3. DeSantis CE, Ma J, Gaudet MM, Newman LA, Miller KD, Goding Sauer A, et al. Breast cancer statistics, 2019. CA: A Cancer Journal for Clinicians 2019;69:438-51.
4. Globocan. Breast. [cited 2024 April]. Available from: https://gco.iarc.fr/today/data/factsheets/cancers/20-Breast-fact-sheet.pdf.

Table S2. Baseline Characteristics in Patients With *ESR1*-Mutated Tumors by Prior ET+CDK4/6i Duration

	Prior ET+CDK4/6i Duration in the Metastatic Setting (N=222)					
	≥6 Months (92.3%)		≥12 Months (71.6%)		≥18 Months (50.0%)	
**Parameter**	**Elacestrant (n=103)**	**SOC (n=102)**	**Elacestrant (n=78)**	**SOC (n=81)**	**Elacestrant (n=55)**	**SOC (n=56)**
Median age, years (range)	64 (28-89)	62.5 (32-83)	65.5 (40-89)	63 (32-82)	67 (40-88)	63 (32-82)
Female, n (%)	103 (100)	102 (100)	78 (100)	81 (100)	55 (100)	56 (100)
Race or ethnicity, n (%)						
Asian	5 (4.9)	7 (6.9)	3 (3.9)	3 (3.7)	3 (5.5)	3 (5.4)
Black or African American	3 (2.9)	4 (3.9)	3 (3.9)	4 (4.9)	2 (3.6)	4 (7.1)
Other	1 (1.0)	0	1 (1.3)	0	1 (1.8)	0
White	77 (74.8)	74 (72.6)	59 (75.6)	59 (72.8)	39 (70.9)	40 (71.4)
Hispanic or Latino	8 (7.8)	8 (7.8)	6 (7.7)	7 (8.6)	4 (7.3)	6 (10.7)
ECOG PS 0, n (%)	60 (58.3)	58 (56.9)	42 (53.9)	49 (60.5)	28 (50.9)	34 (60.7)
Visceral metastases, n (%)	71 (68.9)	72 (70.6)	58 (74.4)	57 (70.4)	42 (76.4)	41 (73.2)
Prior adjuvant therapy, n (%)	53 (51.5)	57 (55.9)	44 (56.4)	47 (58.0)	35 (63.6)	30 (53.6)
No. of prior lines of endocrine therapy in the advanced or metastatic setting, n (%)						
1	65 (63.1)	64 (62.8)	49 (62.8)	55 (67.9)	35 (63.6)	41 (73.2)
2	38 (36.9)	38 (37.3)	29 (37.2)	26 (32.1)	20 (36.4)	15 (26.8)

No. of prior lines of chemotherapy in the advanced or metastatic setting, n (%)						
0	81 (78.6)	74 (72.6)	62 (79.5)	63 (77.8)	43 (78.2)	43 (76.8)
1	22 (21.4)	28 (27.5)	16 (20.5)	18 (22.2)	12 (21.8)	13 (23.2)
Prior CDK4/6 inhibitor, n (%)						
Abemaciclib	4 (3.9)	4 (3.9)	3 (3.8)	3 (3.7)	2 (3.6)	1 (1.8)
Palbociclib	92 (89.3)	93 (91.2)	70 (89.7)	77 (95.1)	50 (90.9)	54 (96.4)
Ribociclib	17 (16.5)	17 (16.7)	14 (17.9)	11 (13.6)	8 (14.5)	8 (14.3)
Any prior endocrine therapy, n (%)	103 (100)	100 (98.0)	78 (100)	80 (98.8)	55 (100)	55 (98.2)
Fulvestrant, n (%)	22 (21.4)	25 (24.5)	13 (16.7)	22 (27.2)	9 (16.4)	16 (28.6)
Aromatase inhibitor, n (%)	96 (93.2)	90 (88.2)	72 (92.3)	71 (87.7)	49 (89.1)	48 (85.7)
Tamoxifen, n (%)	9 (8.7)	9 (8.8)	7 (9.0)	7 (8.6)	5 (9.1)	6 (10.7)
PI3K inhibitor, n (%)	1 (1.0)	0	0	0	0	0
mTOR inhibitor, n (%)	7 (6.8)	3 (2.9)	5 (6.4)	1 (1.2)	5 (9.1)	1 (1.8)

CDK4/6, cyclin-dependent kinase 4/6; ECOG PS, Eastern Cooperative Oncology Group performance status; *ESR1*, estrogen receptor 1; ET+CDK4/6i, endocrine therapy plus cyclin-dependent kinase 4/6 inhibitor; mTOR, mammalian target of rapamycin; PI3K, phosphoinositide 3-kinases; SOC, standard-of-care endocrine therapy (aromatase inhibitor or fulvestrant)

Table S3. PFS in Subgroups of Patients with *ESR1*-mutated Tumors and Prior ET+CDK4/6i ≥12 Months by *PIK3CA* Mutation Location, and *BRCA1/2* Mutation Status

Patient Subgroup	n	Median PFS, months		HR (95% CI)
		Elacestrant	SOC	
ESR1- and *PIK3CA* H1047X-mutated tumors, and ET+CDK4/6i ≥12 months	26	4.6	3.3	0.77 (0.27 – 2.15)
ESR1- and *PIK3CA* E542X/E545X-mutated tumors, and ET+CDK4/6i ≥12 months	27	5.5	1.9	0.80 (0.26 – 2.48)
ESR1- and *BRCA1/2*-mutated tumors and ET+CDK4/6i ≥12 months	32	5.5	2.1	0.44 (0.15 – 1.29)

BRCA1/2, breast cancer genes 1 and/or 2; CI, confidence interval; *ESR1*, estrogen receptor 1; ET+CDK4/6i, endocrine therapy plus cyclin-dependent kinase 4/6 inhibitor; HR, hazard ratio; PFS, progression-free survival; *PIK3CA*, phosphatidylinositol-4,5-bisphosphate 3-kinase catalytic subunit alpha; SOC, standard-of-care endocrine therapy (aromatase inhibitor or fulvestrant)

Table S4: The Most Common Adverse Events (>10%) in Either Treatment Arm in Patients With *ESR1*-Mutated Tumors and Prior ET+CDK4/6i ≥6 Months

Adverse Reaction[a]	Prior ET+CDK4/6i ≥6 Months			
	Elacestrant (n=103)		SOC (n=95)[b]	
	All Grades (%)	Grade ≥3 (%)	All Grades (%)	Grade ≥3 (%)
**Musculoskeletal and connective tissue disorders**				
Musculoskeletal pain[c]	45.6	7.8	36.8	1.1
**Gastrointestinal disorders**				
Nausea	35	3.9	16.8	1.1
Vomiting[c]	18.4	1.9	8.4	0
Diarrhea	16.5	0	11.6	1.1
Constipation	11.7	0	7.4	0
Abdominal pain[c]	8.7	1.9	10.5	1.1
Dyspepsia	11.7	0	3.2	0
**General disorders and administration site conditions**				
Fatigue[c]	27.2	3.9	28.4	1.1
**Metabolism and nutritional disorders**				
Decreased appetite	16.5	1	6.3	1.1
**Nervous system disorders**				
Headache	14.6	1.9	9.5	0
**Psychiatric disorders**				
Insomnia	12.6	1	7.4	1.1
**Investigations**				

Aspartate aminotransferase increased	9.7	1.9	11.6	2.1
Alanine aminotransferase increased	4.9	1	11.6	0
**Blood and lymphatic system disorders**				
Anemia	8.7	1.9	10.5	2.1
**Nausea-related adverse events**				
Dose-reduction rate due to nausea, n (%)	2 (1.9)		0	
Discontinuation rate due to nausea, n (%)	1 (1)		0	
Antiemetic use, n (%)	15 (14.6)		4 (17.4) (AI)<br>3 (4.2) (Fulvestrant)	

[a]Adverse reactions were graded using National Cancer Institute Common Terminology Criteria for Adverse Events version 5.0; [b]7 patients were randomized but did not receive study drug; therefore, they are not included in this subgroup safety population; [c]Includes other related terms. AI, aromatase inhibitor; *ESR1*, estrogen receptor 1; ET+CDK4/6, endocrine therapy plus cyclin-dependent kinase 4/6 inhibitor; SOC, standard-of-care endocrine therapy (aromatase inhibitor or fulvestrant)

Table S5: The Most Common Adverse Events (>10%) in Either Treatment Arm in Patients With *ESR1*-Mutated Tumors and Prior ET+CDK4/6i ≥12 Months

Adverse Reaction[a]	Prior ET+ CDK4/6i ≥12 Months			
	Elacestrant (n=78)		SOC (n=75)[b]	
	All Grades (%)	Grade ≥3 (%)	All Grades (%)	Grade ≥3 (%)
**Musculoskeletal and connective tissue disorders**				
Musculoskeletal pain[c]	50	6.4	37.3	1.3
**Gastrointestinal disorders**				
Nausea	38.5	5.1	14.7	1.3
Vomiting[c]	20.5	2.6	8	0
Diarrhea	20.5	0	12	0
Abdominal pain[c]	10.3	2.6	10.7	1.3
Dyspepsia	12.8	0	4	0
**General disorders and administration site conditions**				
Fatigue[c]	25.6	3.8	22.7	1.3
**Metabolism and nutritional disorders**				
Decreased appetite	15.4	0	6.7	1.3
**Nervous system disorders**				
Headache	16.7	1.3	12	0
**Vascular disorders**				
Hot flush	11.5	0	9.3	0
**Infections and infestations**				
Urinary tract infection	10.3	0	6.7	1.3

**Psychiatric Disorders**	21.8	0	13.3	1.3
Insomnia	10.3	0	8	1.3
**Investigations**				
Aspartate aminotransferase increased	10.3	2.6	14.7	2.7
Alanine aminotransferase increased	5.1	1.3	14.7	0
**Blood and lymphatic system disorders**				
Anemia	9	2.6	12	2.7
**Nausea-related adverse events**				
Dose-reduction rate due to nausea, n (%)	2 (2.6)		0	
Discontinuation rate due to nausea, n (%)	1 (1.3)		0	
Antiemetic use, n (%)	12 (15.4)		4 (20) (AI)<br>2 (3.6) (Fulvestrant)	

[a]Adverse reactions were graded using National Cancer Institute Common Terminology Criteria for Adverse Events version 5.0; [b]6 patients were randomized but did not receive study drug; therefore, they are not included in this subgroup safety population. [c]Includes other related terms; AI, aromatase inhibitor; *ESR1*, estrogen receptor 1; ET+CDK4/6, endocrine therapy plus cyclin-dependent kinase 4/6 inhibitor; SOC, standard-of-care endocrine therapy (aromatase inhibitor or fulvestrant)

Table S6: The Most Common Adverse Events (>10%) in Either Treatment Arm in Patients With *ESR1*-Mutated Tumors and Prior ET+CDK4/6i ≥18 Months

Adverse Reaction[a]	Prior ET+CDK4/6i ≥18 Months			
	Elacestrant (n=55)		SOC (n=50)[b]	
	All Grades (%)	Grade ≥3 (%)	All Grades (%)	Grade ≥3 (%)
**Musculoskeletal and connective tissue disorders**				
Musculoskeletal pain[c]	49.1	7.3	34	0
**Gastrointestinal disorders**				
Nausea	40	7.3	14	2
Vomiting[c]	25.5	3.6	4	0
Diarrhea	23.6	0	10	0
Abdominal pain[c]	12.7	1.8	10	0
Dyspepsia	12.7	0	2	0
**General disorders and administration site conditions**				
Fatigue[c]	27.3	3.6	24	2
**Metabolism and nutritional disorders**				
Decreased appetite	14.5	0	2	0
**Nervous system disorders**				
Headache	20	1.8	8	0
**Vascular disorders**				
Hot flush	12.7	0	10	0
**Psychiatric disorders**				
Insomnia	12.7	0	10	2

**Investigations**				
Aspartate aminotransferase increased	10.9	1.8	16	2
Alanine aminotransferase increased	7.3	1.8	16	0
**Nausea-related adverse events**				
Dose-reduction rate due to nausea, n (%)	2 (3.6)		0	
Discontinuation rate due to nausea, n (%)	1 (1.8)		0	
Antiemetic use, n (%)	10 (18.2)		3 (21.4) (AI)<br>1 (2.8) (Fulvestrant)	

[a]Adverse reactions were graded using National Cancer Institute Common Terminology Criteria for Adverse Events version 5.0; [b]6 patients were randomized but did not receive study drug; therefore, they are not included in this subgroup safety population. [c]Includes other related terms. AI, aromatase inhibitor; *ESR1*, estrogen receptor 1; ET+CDK4/6, endocrine therapy plus cyclin-dependent kinase 4/6 inhibitor; SOC, standard-of-care endocrine therapy (aromatase inhibitor or fulvestrant)

Table S7: The Most Common Adverse Events (>10%) in Either Treatment Arm in Patients With *ESR1*-Mutated Tumors, Prior ET+CDK4/6i ≥12 Months, and Bone Metastases

Adverse Reaction[a]	Bone Metastases			
	Elacestrant (n=67)		SOC (n=64)[b]	
	All Grades, %	Grade ≥3, %	All Grades, %	Grade ≥3, %
**Musculoskeletal and connective tissue disorders**				
Musculoskeletal pain[c]	50.7	4.5	37.5	1.6
**Gastrointestinal disorders**				
Nausea	41.8	3	14.1	1.6
Diarrhea	20.9	0	12.5	0
Vomiting[c]	19.4	1.5	9.4	0
Dyspepsia	11.9	0	3.1	0
Abdominal pain[c]	9	3	10.9	1.6
**General disorders and administration site conditions**				
Fatigue	25.4	1.5	21.9	1.6
Injection site pain	0	0	10.9	0
**Nervous system disorders**				
Headache	19.4	1.5	12.5	0
**Metabolism and nutrition disorders**				
Decreased appetite	16.4	0	7.8	1.6
**Vascular disorders**				
Hot flush	13.4	0	7.8	0
**Psychiatric disorders**				

Insomnia	10.4	0	7.8	0
**Respiratory, thoracic and mediastinal disorders**				
Dyspnea	10.4	0	6.3	0
**Investigations**				
Aspartate aminotransferase increased	10.4	1.5	15.6	3.1
Alanine aminotransferase increased	4.5	1.5	17.2	0
**Blood and lymphatic system disorders**				
Anemia	9	1.5	14.1	3.1
**Nausea-related adverse events**				
Dose-reduction rate due to nausea, n (%)	1 (1.5)		0	
Discontinuation rate due to nausea, n (%)	0		0	
Antiemetic use, n (%)	11 (16.4)		4 (22.2) (AI)<br>2 (4.3) (Fulvestrant)	

[a]Adverse reactions were graded using National Cancer Institute Common Terminology Criteria for Adverse Events version 5.0; [b]5 patients were randomized but did not receive study drug; therefore, they are not included in this subgroup safety population. [c]Includes other related terms. AI, aromatase inhibitor; *ESR1*, estrogen receptor 1; ET+CDK4/6, endocrine therapy plus cyclin-dependent kinase 4/6 inhibitor; SOC, standard-of-care endocrine therapy (aromatase inhibitor or fulvestrant)

Table S8: The Most Common Adverse Events (>10%) in Either Treatment Arm in Patients With *ESR1*-Mutated Tumors, Prior ET+CDK4/6i ≥12 Months, and Liver or Lung Metastases

Adverse Reaction[a]	Liver or Lung Metastases			
	Elacestrant (n=56)		SOC (n=54)[b]	
	All Grades, %	Grade ≥3, %	All Grades, %	Grade ≥3, %
**Musculoskeletal and connective tissue disorders**				
Musculoskeletal pain[c]	50	3.6	29.6	0
**Gastrointestinal disorders**				
Nausea	37.5	7.1	16.7	1.9
Vomiting[c]	23.2	3.6	5.6	0
Diarrhea	21.4	0	11.1	0
Dyspepsia	14.3	0	5.6	0
Abdominal pain[c]	5.4	0	13	1.9
**General disorders and administration site conditions**				
Fatigue	21.4	3.6	29.6	1.9
**Metabolism and nutrition disorders**				
Decreased appetite	14.3	0	5.6	1.9
**Nervous system disorders**				
Headache	12.5	1.8	9.3	0
**Infections and infestations**				
Urinary tract infection	12.5	0	5.6	0
**Investigations**				
Aspartate aminotransferase increased	12.5	3.6	18.5	3.7

Alanine aminotransferase increased	7.1	1.8	14.8	0
**Psychiatric disorders**				
Insomnia	7.1	0	11.1	1.9
**Nausea-related adverse events**				
Dose-reduction rate due to nausea, n (%)	2 (3.6)		0	
Discontinuation rate due to nausea, n (%)	1 (1.8)		0	
Antiemetic use, n (%)	9 (16.1)		4 (26.7) (AI)<br>1 (2.6) (fulvestrant)	

[a]Adverse reactions were graded using National Cancer Institute Common Terminology Criteria for Adverse Events version 5.0; [b]3 patients were randomized but did not receive study drug; therefore, they are not included in this subgroup safety population. [c]Includes other related terms. AI, aromatase inhibitor; *ESR1*, estrogen receptor 1; ET+CDK4/6, endocrine therapy plus cyclin-dependent kinase 4/6 inhibitor; SOC, standard-of-care endocrine therapy (aromatase inhibitor or fulvestrant)

Table S9: The Most Common Adverse Events (>10%) in Either Treatment Arm in Patients With *ESR1*-Mutated Tumors, Prior ET+CDK4/6i ≥12 Months, and <3 Metastatic Sites

Adverse Reaction[a]	<3 Metastatic Sites			
	Elacestrant (n=42)		SOC (n=37)[b]	
	All Grades, %	Grade ≥3, %	All Grades, %	Grade ≥3, %
**Musculoskeletal and connective tissue disorders**				
Musculoskeletal pain[c]	50	9.5	37.8	2.7
**Gastrointestinal disorders**				
Nausea	47.6	2.4	18.9	0
Diarrhea	23.8	0	16.2	0
Abdominal pain[c]	16.7	4.8	13.5	2.7
Vomiting[c]	16.7	0	5.4	0
**General disorders and administration site conditions**				
Fatigue	28.6	4.8	29.7	0
**Nervous system disorders**				
Headache	23.8	0	13.5	0
**Metabolism and nutrition disorders**				
Decreased appetite	16.7	0	5.4	2.7
**Psychiatric disorders**				
Insomnia	16.7	0	8.1	2.7
Anxiety	14.3	0	8.1	0
**Vascular disorders**				
Hot flush	11.9	0	5.4	0

Hypertension	11.9	2.4	2.7	0
**Blood and lymphatic system disorders**				
Anemia	9.5	2.4	10.8	2.7
**Investigations**				
Aspartate aminotransferase increased	9.5	2.4	16.2	5.4
Alanine aminotransferase increased	7.1	2.4	13.5	0
**Infections and infestations**				
Urinary tract infection	7.1	0	10.8	2.7
**Nausea-related adverse events**				
Dose-reduction rate due to nausea, n (%)	1 (2.4)		0	
Discontinuation rate due to nausea, n (%)	0		0	
Antiemetic use, n (%)	7 (16.7)		2 (14.3) (AI)<br>1 (4.3) (Fulvestrant)	

[a]Adverse reactions were graded using National Cancer Institute Common Terminology Criteria for Adverse Events version 5.0; [b]3 patients were randomized but did not receive study drug; therefore, they are not included in this subgroup safety population. [c]Includes other related terms. AI, aromatase inhibitor; *ESR1*, estrogen receptor 1; ET+CDK4/6, endocrine therapy plus cyclin-dependent kinase 4/6 inhibitor; SOC, standard-of-care endocrine therapy (aromatase inhibitor or fulvestrant)

Table S10: The Most Common Adverse Events (>10%) in Either Treatment Arm in Patients With *ESR1*-Mutated Tumors, Prior ET+CDK4/6i ≥12 Months, and ≥3 Metastatic Sites

Adverse Reaction[a]	≥3 Metastatic Sites			
	Elacestrant (n=28)		SOC (n=24)[b]	
	All Grades, %	Grade ≥3, %	All Grades, %	Grade ≥3, %
**Musculoskeletal and connective tissue disorders**				
Musculoskeletal pain[c]	50	3.6	33.3	0
**Gastrointestinal disorders**				
Nausea	32.1	10.7	12.5	0
Vomiting[c]	25	7.1	12.5	0
Dyspepsia	17.9	0	0	0
Diarrhea	14.3	0	8.3	0
**General disorders and administration site conditions**				
Fatigue	25	3.6	16.7	0
Peripheral edema	10.7	0	4.2	0
Injection site pain	0	0	12.5	0
**Metabolism and nutrition disorders**				
Decreased appetite	14.3	0	8.3	0
**Respiratory, thoracic and mediastinal disorders**				
Dyspnea	10.7	0	8.3	0
**Vascular disorders**				
Hot flush	10.7	0	8.3	0
**Blood and lymphatic system disorders**				

Anemia	10.7	3.6	20.8	4.2
**Investigations**				
Aspartate aminotransferase increased	7.1	3.6	16.7	0
Alanine aminotransferase increased	3.6	0	16.7	0
**Nausea-related adverse events**				
Dose-reduction rate due to nausea, n (%)	1 (3.6)		0	
Discontinuation rate due to nausea, n (%)	1 (3.6)		0	
Antiemetic use, n (%)	5 (17.9)		1 (33.3) (AI)<br>1 (4.8) (Fulvestrant)	

[a]Adverse reactions were graded using National Cancer Institute Common Terminology Criteria for Adverse Events version 5.0; [b]1 patient was randomized but did not receive study drug; therefore, they are not included in this subgroup safety population. [c]Includes other related terms. AI, aromatase inhibitor; *ESR1*, estrogen receptor 1; ET+CDK4/6, endocrine therapy plus cyclin-dependent kinase 4/6 inhibitor; SOC, standard-of-care endocrine therapy (aromatase inhibitor or fulvestrant)

Table S11: The Most Common Adverse Events (>10%) in Either Treatment Arm in Patients With *ESR1*-Mutated Tumors, Prior ET+CDK4/6i ≥12 Months, and *PIK3CA* Mutation

Adverse Reaction[a]	*PIK3CA* Mutation			
	Elacestrant (n=27)		SOC (n=32)[b]	
	All Grades, %	Grade ≥3, %	All Grades, %	Grade ≥3, %
**Musculoskeletal and connective tissue disorders**				
Musculoskeletal pain[c]	44.4	0	43.8	3.1
**Gastrointestinal disorders**				
Nausea	37	3.7	18.8	0
Vomiting[c]	29.6	3.7	9.4	0
Diarrhea	14.8	0	12.5	0
Stomatitis	11.1	0	0	0
**Psychiatric disorders**				
Insomnia	22.2	0	3.1	0
**Blood and lymphatic system disorders**				
Anemia	18.5	3.7	25	6.3
**Respiratory, thoracic and mediastinal disorders**				
Dyspnea	18.5	0	9.4	0
**Metabolism and nutrition disorders**				
Decreased appetite	18.5	0	6.3	0
Hyperglycemia	11.1	3.7	3.1	3.1
**Investigations**				
Aspartate aminotransferase increased	18.5	3.7	6.3	0

Gamma-glutamyl transferase increased	11.1	7.4	3.1	0
**General disorders and administration site conditions**				
Fatigue	14.8	0	15.6	0
**Nausea-related adverse events**				
Dose-reduction rate due to nausea, n (%)	0		0	
Discontinuation rate due to nausea, n (%)	0		0	
Antiemetic use, n (%)	5 (18.5)		2 (22.2) (AI)<br>1 (4.3) (Fulvestrant)	

[a]Adverse reactions were graded using National Cancer Institute Common Terminology Criteria for Adverse Events version 5.0; [b]3 patients were randomized but did not receive study drug; therefore, they are not included in this subgroup safety population. [c]Includes other related terms. AI, aromatase inhibitor; *ESR1*, estrogen receptor 1; ET+CDK4/6, endocrine therapy plus cyclin-dependent kinase 4/6 inhibitor; PIK3CA, phosphatidylinositol-4,5-bisphosphate 3-kinase catalytic subunit alpha; SOC, standard-of-care endocrine therapy (aromatase inhibitor or fulvestrant)

Table S12: The Most Common Adverse Events (>10%) in Either Treatment Arm in Patients With *ESR1*-Mutated Tumors, Prior ET+CDK4/6i ≥12 Months, and *TP53* Mutation

Adverse Reaction[a]	*TP53* Mutation			
	Elacestrant (n=32)		SOC (n=27)[b]	
	All Grades, %	Grade ≥3, %	All Grades, %	Grade ≥3, %
**Musculoskeletal and connective tissue disorders**				
Musculoskeletal pain[c]	43.8	6.3	25.9	0
Myalgia	12.5	0	7.4	0
**Gastrointestinal disorders**				
Nausea	28.1	0	14.8	0
Diarrhea	15.6	0	11.1	0
Dyspepsia	15.6	0	7.4	0
Vomiting[c]	15.6	0	3.7	0
Abdominal pain[c]	12.5	6.3	7.4	0
**General disorders and administration site conditions**				
Fatigue	28.1	3.1	29.6	0
**Nervous system disorders**				
Headache	18.8	3.1	11.1	0
**Metabolism and nutrition disorders**				
Decreased appetite	15.6	0	3.7	0
**Vascular disorders**				
Hot flush	12.5	0	7.4	0
**Infections and infestations**				

Urinary tract infection	12.5	0	7.4	0
**Investigations**				
Aspartate aminotransferase increased	12.5	0	14.8	7.4
Blood cholesterol increased	12.5	0	0	0
Alanine aminotransferase increased	6.3	0	14.8	0
**Respiratory, thoracic and mediastinal disorders**				
Dyspnea	6.3	0	11.1	0
**Blood and lymphatic system disorders**				
Anemia	3.1	0	11.1	3.7
**Nausea-related adverse events**				
Dose-reduction rate due to nausea, n (%)	0		0	
Discontinuation rate due to nausea, n (%)	0		0	
Antiemetic use, n (%)	5 (15.6)		0	

[a]Adverse reactions were graded using National Cancer Institute Common Terminology Criteria for Adverse Events version 5.0; [b]2 patients were randomized but did not receive study drug; therefore, they are not included in this subgroup safety population. [c]Includes other related terms. AI, aromatase inhibitor; *ESR1*, estrogen receptor 1; ET+CDK4/6, endocrine therapy plus cyclin-dependent kinase 4/6 inhibitor; SOC, standard-of-care endocrine therapy (aromatase inhibitor or fulvestrant); *TP53*, tumor protein p53 gene

Table S13: The Most Common Adverse Events (>10%) in Either Treatment Arm in Patients With *ESR1*-Mutated Tumors, Prior ET+CDK4/6i ≥12 Months, and HER2-Low Expression

Adverse Reaction[a]	HER2-low Expression			
	Elacestrant (n=37)		SOC (n=36)[b]	
	All Grades, %	Grade ≥3, %	All Grades, %	Grade ≥3, %
**Musculoskeletal and connective tissue disorders**				
Musculoskeletal pain[c]	45.9	0	38.9	0
**Gastrointestinal disorders**				
Nausea	40.5	5.4	5.6	2.8
Diarrhea	27	0	11.1	0
Vomiting[c]	18.9	2.7	2.8	0
Dyspepsia	10.8	0	0	0
**General disorders and administration site conditions**				
Fatigue	27	0	19.4	2.8
**Nervous system disorders**				
Headache	16.2	2.7	8.3	0
**Psychiatric disorders**				
Anxiety	10.8	0	0	0
Insomnia	10.8	0	5.6	0
**Vascular disorders**				
Hypertension	10.8	2.7	5.6	2.8
Hot flush	8.1	0	11.1	0
**Blood and lymphatic system disorders**				

Anemia	8.1	0	11.1	2.8
**Investigations**				
Alanine aminotransferase increased	2.7	2.7	11.1	0
**Nausea-related adverse events**				
Dose-reduction rate due to nausea, n (%)	1 (2.7)		0	
Discontinuation rate due to nausea, n (%)	0		0	
Antiemetic use, n (%)	4 (10.8)		1 (11.1) (AI)<br>2 (7.4) (Fulvestrant)	

[a]Adverse reactions were graded using National Cancer Institute Common Terminology Criteria for Adverse Events version 5.0; [b]4 patients were randomized but did not receive study drug; therefore, they are not included in this subgroup safety population. [c]Includes other related terms. AI, aromatase inhibitor; *ESR1*, estrogen receptor 1; ET+CDK4/6, endocrine therapy plus cyclin-dependent kinase 4/6 inhibitor; HER2, human epidermal growth factor 2; SOC, standard-of-care endocrine therapy (aromatase inhibitor or fulvestrant)

Table S14: The Most Common Adverse Events (>10%) in Either Treatment Arm in Patients With *ESR1* D538G-Mutated Tumors, and Prior ET+CDK4/6i ≥12 Months

Adverse Reaction[a]	*ESR1* D538G Variant			
	Elacestrant (n=48)		SOC (n=47)[b]	
	All Grades, %	Grade ≥3, %	All Grades, %	Grade ≥3, %
**Musculoskeletal and connective tissue disorders**				
Musculoskeletal pain[c]	58.3	6.3	36.2	0
**Gastrointestinal disorders**				
Nausea	45.8	6.3	10.6	0
Diarrhea	22.9	0	8.5	0
Vomiting[c]	16.7	4.2	4.3	0
Dyspepsia	12.5	0	6.4	0
Abdominal pain[c]	8.3	0	10.6	0
**General disorders and administration site conditions**				
Fatigue	20.8	4.2	21.3	0
Pyrexia	10.4	0	2.1	0
Injection site pain	0	0	12.8	0
**Metabolism and nutrition disorders**				
Decreased appetite	16.7	0	4.3	0
**Vascular disorders**				
Hot flush	14.6	0	10.6	0
**Investigations**				
Aspartate aminotransferase increased	14.6	4.2	8.5	2.1

Gamma-glutamyltransferase increased	10.4	4.2	4.3	2.1
**Respiratory, thoracic and mediastinal disorders**				
Dyspnea	12.5	0	4.3	0
**Blood and lymphatic system disorders**				
Anemia	12.5	4.2	8.5	2.1
**Nervous system disorders**				
Headache	12.5	0	12.8	0
**Infections and infestations**				
Urinary tract infection	10.4	0	6.4	2.1
**Psychiatric disorders**				
Insomnia	6.3	0	10.6	2.1
**Nausea-related adverse events**				
Dose-reduction rate due to nausea, n (%)	1 (2.1)		0	
Discontinuation rate due to nausea, n (%)	1 (2.1)		0	
Antiemetic use, n (%)	8 (16.7)		1 (10) (AI)<br>1 (2.7) (Fulvestrant)	

[a]Adverse reactions were graded using National Cancer Institute Common Terminology Criteria for Adverse Events version 5.0; [b]2 patients were randomized but did not receive study drug; therefore, they are not included in this subgroup safety population. [c]Includes other related terms. AI, aromatase inhibitor; *ESR1*, estrogen receptor 1; ET+CDK4/6, endocrine therapy plus cyclin-dependent kinase 4/6 inhibitor; SOC, standard-of-care endocrine therapy (aromatase inhibitor or fulvestrant)

Table S15: The Most Common Adverse Events (>10%) in Either Treatment Arm in Patients With *ESR1* Y537S/N-Mutated Tumors, and Prior ET+CDK4/6i ≥12 Months

Adverse Reaction[a]	*ESR1* Y537S/N Variants			
	Elacestrant (n=49)		SOC (n=39)[b]	
	All Grades, %	Grade ≥3, %	All Grades, %	Grade ≥3, %
**Musculoskeletal and connective tissue disorders**				
Musculoskeletal pain[c]	53.1	4.1	28.2	2.6
**Gastrointestinal disorders**				
Nausea	38.8	4.1	15.4	0
Vomiting[c]	22.4	2	7.7	0
Diarrhea	18.4	0	7.7	0
Dyspepsia	14.3	0	2.6	0
Abdominal pain[c]	12.2	2	7.7	0
**General disorders and administration site conditions**				
Fatigue	34.7	4.1	17.9	0
Peripheral edema	10.2	0	5.1	0
Injection site pain	0	0	10.3	0
**Nervous system disorders**				
Headache	20.4	2	7.7	0
**Metabolism and nutrition disorders**				
Decreased appetite	18.4	0	7.7	0
**Psychiatric disorders**				
Insomnia	14.3	0	5.1	0

Anxiety	10.2	0	5.1	0
**Infections and infestations**				
Urinary tract infection	12.2	0	5.1	0
**Investigations**				
Aspartate aminotransferase increased	12.2	4.1	15.4	2.6
Alanine aminotransferase increased	8.2	2	17.9	0
**Vascular disorders**				
Hot flush	10.2	0	7.7	0
Hypertension	10.2	2	2.6	0
**Respiratory, thoracic and mediastinal disorders**				
Dyspnea	10.2	0	7.7	0
**Blood and lymphatic system disorders**				
Anemia	8.2	0	17.9	5.1
**Nausea-related adverse events**				
Dose-reduction rate due to nausea, n (%)	1 (2)		0	
Discontinuation rate due to nausea, n (%)	0		0	
Antiemetic use, n (%)	9 (18.4)		3 (33.3) (AI)<br>2 (6.7) (Fulvestrant)	

[a]Adverse reactions were graded using National Cancer Institute Common Terminology Criteria for Adverse Events version 5.0; [b]4 patients were randomized but did not receive study drug; therefore, they are not included in this subgroup safety population. [c]Includes other related terms. AI, aromatase inhibitor; *ESR1*, estrogen receptor 1; ET+CDK4/6, endocrine therapy plus cyclin-dependent kinase 4/6 inhibitor; SOC, standard-of-care endocrine therapy (aromatase inhibitor or fulvestrant)
